# Varicella-zoster virus seroprevalence in children and adolescents in the pre-varicella vaccine era, Germany

**DOI:** 10.1186/s12879-017-2461-2

**Published:** 2017-05-19

**Authors:** Miriam Wiese-Posselt, Anette Siedler, Annette Mankertz, Andreas Sauerbrei, Hartmut Hengel, Ole Wichmann, Christina Poethko-Müller

**Affiliations:** 1 Department for Infectious Disease Epidemiology, Robert Koch Institute, Immunization Unit, Seestrasse 10, 13353 Berlin, Germany; 2 Department of Infectious Diseases, Robert Koch Institute, Berlin, Germany; 3 Institute of Virology and Antiviral Therapy, German Consulting Laboratory for HSV and VZV, Jena University Hospital, Friedrich-Schiller-University of Jena, Jena, Germany; 4 Institute of Virology, Medical Center, Albrecht-Ludwigs-University, Faculty of Medicine, Freiburg, Germany; 5 Department of Epidemiology and Health Monitoring, Robert Koch Institute, Berlin, Germany

**Keywords:** Varicella-zoster virus, Seroprevalence, Elisa, FAMA, Varicella vaccination

## Abstract

**Background:**

In 2004, universal childhood varicella vaccination was introduced in Germany. We aimed to determine the age-specific prevalence of anti-varicella zoster virus (VZV) IgG-antibodies among children in the pre-varicella vaccine era in Germany, to identify factors associated with VZV seropositivity, and to assess the suitability of a commercially available ELISA for VZV seroepidemiological studies by comparing it with an in-house fluorescent antibody to membrane antigen test (FAMA) as the gold standard.

**Methods:**

Serum samples of 13,433 children and adolescents aged 1–17 years included in the population-based German Health Interview and Examination Survey for Children and Adolescents (KiGGS; conducted 2003–2006) were tested for anti-VZV IgG by ELISA. All samples with equivocal ELISA results and a random selection of ELISA-negative and -positive samples were tested by FAMA. Statistical analyses were conducted using a weighting factor adjusting the study population to the total population in Germany. Seroprevalences were calculated as percentages (%) with a 95% confidence interval (CI). Odds ratios (OR) were computed by multivariate logistic regression to determine the association between socio-demographic factors and VZV seropositivity.

**Results:**

The VZV seropositivity rate was 80.3% (95% CI: 79.3–81.3) in varicella-unvaccinated children and adolescents. VZV seropositivity rates differed significantly between age groups up to age 6 years, but not by gender. Of 118 retested serum samples with an equivocal ELISA result, 45.8% were FAMA-positive. The proportion of samples tested as false-negative in by ELISA varied by age group: 2.6% in children aged 1–6 and 9% in children aged 7–17 years. Multivariate analyses showed that age, having older siblings, and early daycare start were associated with seropositivity in preschoolers; migration background reduced the chance of VZV seropositivity in schoolchildren (OR: 0.65; 0.43–0.99) and adolescents (OR: 0.62; 0.4–0.97).

**Conclusion:**

In the pre-varicella vaccine era, most children in Germany contracted varicella by age six. Schoolchildren with a migration background and children without siblings have an increased risk of being VZV seronegative and should be targeted for catch-up vaccination, if they have no history of chickenpox. ELISAs are suitable for use in population-level serosurveys on VZV, but samples with equivocal ELISA results should be retested by FAMA.

## Background

Varicella-zoster virus (VZV) is a ubiquitous human herpesvirus. Primary infection with VZV results in varicella (chickenpox), which mostly affects children and is regarded as a generally benign illness [1]. However, varicella can also lead to serious complications resulting in hospitalization or even death [2]. Particularly in adults or in immunocompromised patients, varicella can take a severe course. After primary infection, immunity against varicella is considered to protect life-long. However, VZV becomes latent in the dorsal root ganglia; and later in life, VZV reactivation can cause herpes zoster (shingles).

In 2004, the German Standing Committee on Vaccination (STIKO) recommended universal varicella vaccination for children with a single dose to be given at the age of 11–14 months [3]. In 2009, a two-dose schedule was endorsed with a second dose at the age of 15–23 months [4]. In the birth cohorts of 2004 to 2009, varicella vaccination coverage increased in 24-month-old children from 43% to 87% for the first dose and from 1% to 64% for the second dose [5]. With the increase in coverage, a significant decrease in varicella cases was observed in a nationwide physician-based sentinel surveillance system: from April 2005 to March 2012, an 85% decline in varicella cases was reported by sentinel physicians. Furthermore, from April 2005 to March 2009, complications or severe courses of varicella declined by 81% [6, 7]. Based on sentinel and coverage data, vaccine effectiveness was estimated to be 87% after one vaccine dose and 97% after two vaccine doses [8].

For the evaluation of the current universal varicella vaccination strategy, disease burden data before and after vaccine introduction as well as data on vaccination uptake are essential [9]. Seroepidemiological data before and after vaccine introduction serve as an additional pillar in the evaluation of the vaccination strategy and can guide recommendations on catch-up vaccination activities. Although enzyme-linked immunosorbent assays (ELISAs) are the most commonly used test for measuring immunity against VZV (anti-VZV IgG antibody levels), the fluorescent antibody to membrane antigen test (FAMA) is considered to be the “gold standard” assay for determining immunity against VZV, however, FAMA is more labor-intensive and time-consuming [10]. Studies directly comparing these two test methods are rare.

The objectives of this study were: (1) to representatively describe the age-specific prevalence of anti-VZV IgG antibodies for children and adolescents in Germany (1–17 years of age) in the pre-varicella vaccine era, (2) to identify social factors that are associated with the acquisition of anti-VZV IgG antibodies by naturally acquired varicella, and (3) to compare the commercially available standard ELISA versus an in-house FAMA with respect to its suitability as a test for use in population-level serosurveys.

## Methods

### Study population

From May 2003 to May 2006, the Robert Koch Institute conducted the German Health Interview and Examination Survey for Children and Adolescents (KiGGS), which was designed as a population-based, nationally representative cross-sectional study with 17,641 participants (8985 boys and 8656 girls). One participant withdrew the written consent, and thus 17,640 participants were included in KiGGS. The sampling methods used in this survey have been described elsewhere [11]. In addition to interviews with all parents and children (≥11 years old) on demographics, socialization, and health topics, the vaccination status of each child was documented, and a physical examination of each child was performed. Furthermore, blood and urine samples were collected in 1- to 17-year-old children. Serum samples were stored at −20 °C and subsequently tested for antibodies against various pathogens. The KiGGS survey was approved by the Federal Office for Data Protection and by the ethics committee of Charité University Medicine, Berlin, Germany.

### Serological testing

In 2012 and 2013, 13,433 serum samples were tested for anti-VZV IgG by ELISA. Children vaccinated at least once against varicella were included in the analysis for the overall seroprevalence, but the analysis was stratified by vaccination status in order to allow an assessment of the pre-varicella vaccine era data. In a second step, samples that presented equivocal anti-VZV IgG results when tested by ELISA were retested by FAMA. Samples that presented anti-VZV IgG-negative results in children ≥6 years of age were also retested by FAMA. For children <6 years of age, we selected a random subset of sera to be tested by FAMA since the proportion of children who were anti-VZV IgG-negative in this age group was expected to be much higher. The analysis of the first 1000 serum samples tested by ELISA already indicated that younger children have a lower antibody prevalence, which increased to above 80% in children aged 6 years and older. Furthermore, we also used FAMA to test 197 randomly selected serum samples that had shown a positive result in standard ELISA testing.

#### Enzyme-linked immunosorbent assay (ELISA)

The presence of anti-VZV IgG antibodies in each sample was determined using the EUROIMMUN Anti-VZV (IgG) ELISA kit and the EUROIMMUN Analyzer I-2P (Lübeck, Germany). According to the manufacturer’s information, anti-VZV IgG concentrations of ≥110 IU/l, ≥ 80 to <110 IU/l, and <80 IU/l were classed as positive, equivocal, and negative results, respectively [12]. The ELISA tests were performed by the National Reference Center Measles, Mumps, Rubella, which is located at the Robert Koch Institute, Berlin, Germany.

#### Fluorescent antibody to membrane antigen test (FAMA)

In our study, FAMAs were conducted using an in-house modification of the standard version described elsewhere [13]. VZV immunity was determined by measuring the level of fluorescent anti-human IgG that was bound to VZV IgG antibodies linked to viral envelope glycoproteins on the surface of VZV-infected fibroblasts. Serum samples with FAMA titers of ≥1:2 were considered as positive for immunity against VZV; in other words, these anti-VZV IgG sera levels correlate with protection against varicella infection. Detailed information on the in-house FAMA protocol used in this study has been described previously by Sauerbrei et al. [14]. The FAMAs were carried out at the German Consulting Laboratory for HSV and VZV, Jena University Hospital, Friedrich-Schiller-University of Jena, Germany.

### Statistical analysis

Analyses of epidemiological and laboratory data were performed using a weighting factor that adjusted the distribution of the study population from which a blood sample could be taken, according age group, sex, residence in the eastern or western part of Germany or in Berlin, and according nationality to match the distribution of these characteristics in the total population in Germany as of 31 December 2004 [11]. Weighted seroprevalence data are reported as percentages (%) with a 95% confidence interval (CI). ELISA data were stratified by age, gender, and varicella vaccination status. Significant differences between the gender and age groups were assumed if the 95% CIs did not overlap. The test qualities of ELISA and FAMA were compared descriptively on the basis of non-weighted seroprevalence data. For risk factor analyses, VZV seropositivity functioned as the dependent variable and was defined as a positive ELISA result, or as a positive FAMA result in cases when the ELISA had shown an equivocal result. The weighted data set was used. Multivariate logistic regression analyses for complex samples were used to determine possible factors associated with VZV seropositivity (inclusion of variables was guided by hypotheses: age, gender, older and younger siblings living in the same household, migration status, social economic status (SES) and age at start of day-care attendance). The social status score was computed based on three components: parents’ educational and professional statuses as well as the total income available to the family household [15]. Analyses were performed with SPSS version 20 (SPSS Inc., Chicago, Illinios, USA).

## Results

**Fig. 1:**
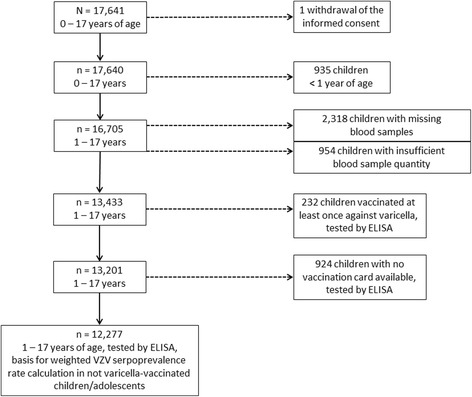
Flow chart indicating the total number of children in the study sample (sera collected 2003–2006, *N* = 17,641) and number of children eventually tested for anti-VZV IgG antibodies by ELISA

**Fig. 2:**
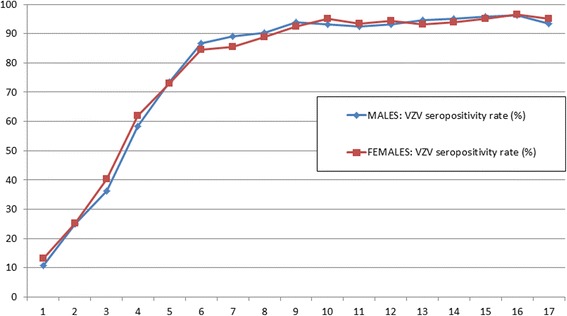
Weighted VZV seropositivity rate stratified by age and sex in varicella-unvaccinated children 1–17 years of age, Germany (sera collected 2003–2006, *n* = 12,277)

Of the 17,640 KiGGS participants, 13,433 children and adolescents that were 1–17 years of age were included in the VZV seroprevalence study. Of those excluded from this study, 935 of the children were <1 year of age and blood sampling was not performed in this age group, 2318 of the children had no available serum sample, and 954 of the children had a serum samples of insufficient quantity (Fig. 1). Gender was distributed evenly in the study population with 6881 (51%) males and 6552 (49%) females. Each age group (1 to 17 years), had 437 to 938 participants included. The mean age of all study participants was 10.1 years (median age: 10.3 years). Of the 13,433 children included, 232 (1.7%) had a vaccination card with at least one vaccination against varicella documented, while 924 (6.9%) children had no vaccination card available. In Table 1, the weighted VZV seroprevalence data are shown stratified by varicella vaccination status. Of the 13,433 sera tested by ELISA, 11,091 (estimated weighted overall VZV seroprevalence rate: 80.3%) samples were classified as positive, 120 (0.9%) samples presented an equivocal result, and 2222 (18.8%) samples were found to be negative for anti-VZV IgG (Table 1). Varicella-vaccinated children and children with no vaccination card available were excluded from further analyses that focused on the seroprevalence and risk of being varicella susceptible in the varicella-unvaccinated population. In Table 2, weighted VZV seropositivity rates detected by ELISA are given for each age group in varicella-unvaccinated children. While VZV seropositivity increased with age with significant differences between single age groups up to the age of 6 years, no significant difference in VZV seropositivity was detected between males and females (Fig. 2).

**Table 1:** Weighted VZV seroprevalence data tested by ELISA and stratified by varicella vaccination status in children 1–17 years of age, Germany (sera collected 2003–2006, *n* = 13,433)

Anti-VZV IgG antibodies tested by ELISA		Varicella vaccination status			Total
		No vaccination card available	Varicella vaccinated	Not varicella vaccinated	
Positive	number (*n*)	811	124	10,156	11,091
	weighted rate (%)	87.7%	48.9%	80.3%	80.3%
	95% CI [%]	[84.8–90.1]	[40.2–57.8]	[79.3–81.3]	[79.4–81.2]
Negative	number (*n*)	109	90	2023	2222
	weighted rate (%)	12.0%	41.4%	18.9%	18.8%
	95% CI [%]	[9.7–14.8]	[34.4–48.8]	[18.0–19.9]	[17.9–19.8]
Equivocal result	number (*n*)	4	18	98	120
	weighted rate (%)	0.3%	9.6%	0.7%	0.9%
	95% CI [%]	[0.1–1.0]	[5.7–15.9]	[0.6–0.9]	[0.7–1.1]
Total	number (*n*)	924	232	12,277	13,433
	weighted rate (%)	100.0%	100.0%	100.0%	100.0%

**Table 2:** Weighted VZV seroprevalence rates stratified by age in varicella-unvaccinated children 1–17 years of age, Germany

Anti-VZV IgG antibodies tested by ELISA, weighted seroprevalence rate [% (95%CI)]	Age in years								
	1 (*n* = 374)	2 (*n* = 503)	3 (*n* = 569)	4 (*n* = 644)	5 (*n* = 662)	6 (*n* = 765)	7 (*n* = 800)	8 (*n* = 832)	9 (*n* = 839)
Positive	12.0 (8.1–17.4)	25.2 (20.8–30.1)	38.4 (33.5–43.5)	60.1 (55.6–64.4)	73.3 (68.9–77.2)	85.7 (82.4–88.5)	87.3 (84.4–89.8)	89.6 (87.2–91.6)	93.2 (90.5–95.1)
Negative	87.8 (82.4–91.7)	74.4 (69.5–78.8)	60.9 (55.8–65.7)	39.4 (35.1–43.9)	26.2 (22.3–30.6)	14.0 (11.2–17.3)	12.0 (9.6–14.9)	9.4 (7.5–11.7)	6.0 (4.2–8.6)
Equivocal result	0.2 (0.0–1.5)	0.4 (0.1–1.5)	0.7 (0.2–2.8)	0.5 (0.2–1.5)	0.5 (0.2–1.4)	0.3 (0.1–0.8)	0.6 (0.3–1.6)	1.0 (0.5–2.0)	0.8 (0.4–1.7)
Anti-VZV IgG antibodies tested by ELISA, weighted seroprevalence rate [% (95%CI)]	Age in years								
	10 (*n* = 808)	11 (*n* = 861)	12 (*n* = 808)	13 (*n* = 830)	14 (*n* = 814)	15 (*n* = 794)	16 (*n* = 712)	17 (*n* = 662)	Total (*n* = 12,277)
Positive	94.1 (91.7–95.9)	93.0 (90.5–94.9)	93.7 (91.4–95.5)	94.0 (91.6–95.7)	94.5 (92.6–96.0)	95.4 (93.5–96.8)	96.4 (94.6–97.6)	94.2 (91.8–96.0)	80.3 (79.3–81.3)
Negative	5.4 (3.8–7.7)	6.6 (4.8–9.0)	5.0 (3.5–7.1)	5.0 (3.5–7.2)	4.5 (3.2–6.3)	3.7 (2.5–5.5)	3.3 (2.1–5.0)	4.3 (2.8–6.6)	18.9 (18.0–19.9)
Equivocal result	0.5 (0.2–1.2)	0.4 (0.1–1.0)	1.3 (0.7–2.5)	1.0 (0.5–2.3)	1.0 (0.5–1.9)	0.8 (0.4–1.9)	0.4 (0.1–1.2)	1.5 (0.7–2.9)	0.7 (0.6–0.9)

Sera collected 2003–2006, *n* = 12,277, tested by ELISA

**Fig. 3:**
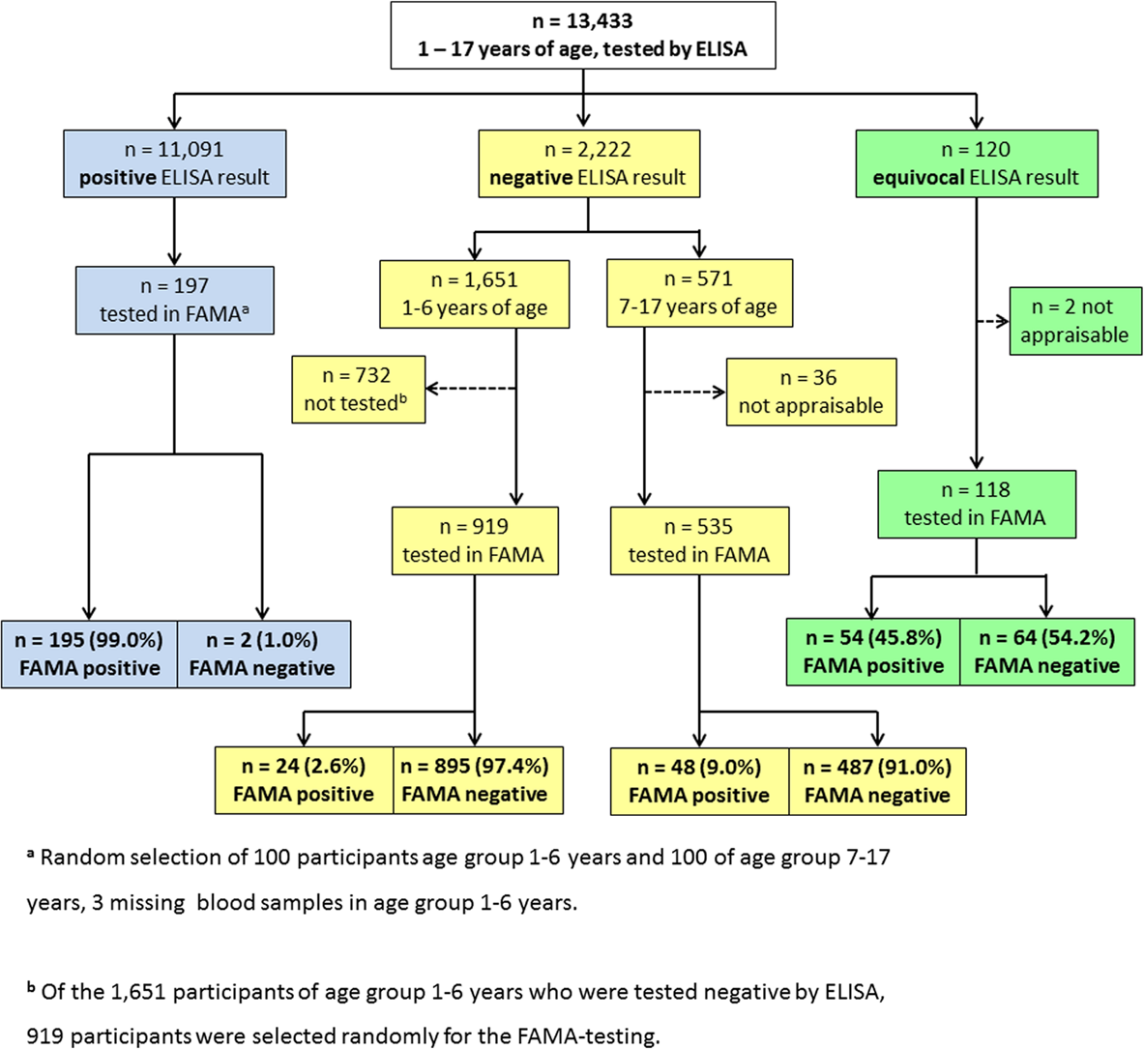
Flow chart of study subjects for VZV seroprevalence testing by FAMA in children 1–17 years of age, Germany (sera collected 2003–2006, *n* = 13,433)

Of the 2222 serum samples that tested negative for anti-VZV IgG by ELISA, 919 samples of children aged 1–6 years and 535 samples of children aged 7–17 years were subsequently examined by FAMA. This test found 24 (2.6%) samples in the age group of 1–6 years and 48 (9.0%) samples in the age group of 7–17 years to be positive. Additionally, 118 of the 120 serum samples that presented an equivocal result by ELISA were tested by FAMA. Of these, 54 (45.8%) samples were assessed as positive by this test. Of the 197 serum samples taht demonstrated a positive ELISA result, 195 (99%) were also FAMA-positive, while only 2 (1%) samples were assessed as negative by FAMA (Fig. 3). In Table 3, we present stratified ELISA and FAMA results by varicella vaccination status. Serum samples from varicella-vaccinated children that had produced either negative or equivocal ELISA results were more frequently assessed as positive by FAMA than were serum samples from varicella-unvaccinated children.

**Table 3:** Results of ELISA and FAMA for the detection of anti-VZV IgG antibodies in sera of children 1–17 years of age (*n* = 1769 tested by ELISA and FAMA). The data set was stratified by the varicella vaccination status of the children. Germany

ELISA positive			
	FAMA		
	positive *n* (%)	negative *n* (%)	total *n* (%)
varicella vaccinated	5 (100%)	0	5 (100%)
not varicella vaccinated	176 (99%)	2 (1%)	178 (100%)
no vaccination card	14 (100%)	0	14 (100%)
total	195 (99%)	2 (1%)	197 (100%)
ELISA negative			
	FAMA		
	positive *n* (%)	negative *n* (%)	total *n* (%)
varicella vaccinated	12 (25.5%)	35 (74.5%)	47 (100%)
not varicella vaccinated	53 (4.0%)	1268 (96.0%)	1321 (100%)
no vaccination card	7 (8.1%)	79 (91.9%)	86 (100%)
total	72 (5.0%)	1382 (95.0%)	1454 (100%)
ELISA equivocal result			
	FAMA		
	positive *n* (%)	negative *n* (%)	total *n* (%)
varicella vaccinated	12 (66.7%)	6 (33.3%)	18 (100%)
not varicella vaccinated	39 (40.6%)	57 (59.4%)	96 (100%)
no vaccination card	3 (75.0%)	1 (25.0%)	4 (100%)
total	54 (45.8%)	64 (54.2%)	118 (100%)

In a logistic regression for complex samples, we did not observe any significant association between VZV seropositivity and either the socioeconomic status or the sex of the child. In children who were 1–10 years of age, increasing age was significantly associated with the acquisition of anti-VZV IgG antibodies. In 1- to 6-year-old children, the presence of older siblings in the same household was related to a higher probability of VZV seropositivity, and in children 7–17 years of age, younger siblings or siblings of the same age functioned as risk factor for VZV seropositivity. Thus, children without siblings in the same household were more likely to be anti-VZV IgG-negative and, therefore, susceptible to contracting chickenpox. Children aged 7–17 years whose parents both had a migration background showed a significantly lower likelihood of VZV seropositivity than children without a migration background or with a one-sided migration background. The start of day-care attendance at younger ages was also significantly associated with VZV seropositivity (Table 4).

**Table 4:** Results of the logistic regression model with VZV seropositivity as the dependent variable; data set stratified in three age groups: children 1 to 6 years of age, 7 to 10, and 11 to 17 years of age. Variables with statistically significant results presented here. Germany (sera collected 2003–2006, available for logistic regression model *n* = 11,374)

	Age 1 to 6 years		Age 7 to 10 years		Age 11 to 17 years	
	*n* = 3162		*n* = 3058		*n* = 5154	
Variable	OR	95% CI	OR	95% CI	OR	95% CI
Age 1 year	Referent					
2 years	2.2	1.4–3.7				
3 years	4.1	2.5–6.9				
4 years	11.0	6.6–18.3				
5 years	21.7	12.8–37.1				
6 years	51.0	30.6–85.3				
7 years			Referent			
8 years			1.4	1.0–1.9		
9 years			2.0	1.3–3.1		
10 years			2.5	1.6–4.1		
Siblings living in the same household:						
only child	Referent	Referent	Referent	Referent	Referent	Referent
younger or same age of the siblings	1.1	0.9–1.5	1.7	1.1–2.6	1.7	1.2–2.4
older siblings	2.3	1.9–2.9	1.3	0.9–1.8	1.2	0.8–1.8
Age at start of day-care attendance (1 to 10 year olds)						
< 1 year	1.6	1.0–2.5	1.8	0.7–4.2		
1 year	1.6	1.1–2.2	2.0	1.1–3.5		
2 years	1.3	0.9–1.8	4.1	2.2–7.7		
> = 3 years	0.9	0.7–1.2	1.7	1.2–2.4		
Migration background:						
none	1.3	0.9–1.7	1.5	1.0–2.3	1.6	1.0–2.5
one-sided	1.1	0.7–1.7	1.3	0.6–2.5	1.5	0.8–3.0
two-sided	Referent		Referent		Referent	

## Discussion

Here, we report for the first time representative population-based VZV seroprevalence data for children aged 1 to 17 years in the pre-varicella vaccine era in Germany. VZV seropositivity increased with age, with 60% of children testing positive at 4 years of age and >90% testing positive at 9 years of age. This gradual increase in VZV seropositivity by age is comparable with the VZV seroprevalence data of German children <18 years of age that were reported by Wutzler et al. in 2001 [13]. For this earlier seroprevalence study, two serum banks were used with residual serum samples that had originally been collected for routine laboratory diagnostics or within the German National Health Interview and Examination Survey between 1995 and 1999. They found that VZV seropositivity was 62% in the 4- to 5-year old children and >90% in the 10- to 11-year old children. Thus, the seroprevalence data of the 1995–1999 study are similar to ours, and we can assume that there was no significant change in the VZV seroprevalence in children in Germany for the time period between 1995 and 1999 and 2003–2006. Also similar to our results, the majority of varicella infection occurred in early childhood in Northern European countries without universal varicella vaccination [16]. Interestingly, children in the Netherlands acquired anti-VZV IgG antibodies, on average, even earlier in childhood than did children in Germany or in Nordic countries, and some have proposed that the relatively high population density in the Netherlands might explain this observation [17]. There are contradicting reports from the Netherlands concerning whether or not VZV seropositivity is associated with day-care attendance [17, 18]. In our study, we found an association between VZV seropositivity and an early (<3 years of age) start of day care attendance. However, this finding may be explained by the low rate of young children (0–3 years of age) who attend day care facilities at all in Germany. In 2006, around 12% of children who were <3 years of age visited a day care center in Germany [19]; in contrast, around 50% of children in the Netherlands who were <3 years of age attended day care facilities in 2010 [20]. Since we can assume that in the absence of varicella vaccination the force of varicella infection was high in day care facilities, we were able to discriminate in regard to VZV seropositivity between children with an early start of day care attendance and children who stayed at home. Additionally, we showed that siblings living in the same household acted as a risk factor for acquiring varicella: for young children, older siblings in the same household were a risk factor for VZV seropositivity, and, for older children, their younger siblings played this role. Thus, in the absence of universal varicella vaccination in children, our data confirm that children are the major force of varicella infection.

There are concerns about an upward shift in varicella infection age following the introduction of universal varicella vaccination in young children [21]. Our study adds to the efforts of monitoring the impact of varicella vaccination in Germany, in part to address these concerns. Despite these worries, this phenomenon has not been observed in varicella surveillance in Germany, which is a physician-based sentinel system established in 2005 shortly after the initiation of universal childhood varicella vaccination [22]. Similarly, in the United States, where universal varicella vaccination in children was implemented in 1995, no shift in varicella cases towards older age groups has been observed as of today [23]. This present serosurvey will serve as a baseline assessment, and a follow-up survey (KiGGS wave 2) which will allow a comparison between more recent results and those of this present study, is currently underway and will recruit children from 2014 to 2017.

In our survey, we detected subgroups within the general population that have an increased risk of being susceptible to varicella infection if they are not reached by an ad-hoc catch-up vaccination; children 7–17 years of age with a migration background and children without siblings were less likely to be anti-VZV IgG-positive. Since no concerted catch-up campaign has been implemented among older children or adolescents following the introduction of universal childhood vaccination in toddlers, pediatricians and general practitioners should be aware of this fact and provide individual varicella catch-up vaccinations to these risk groups if an individual lacks a history of chickenpox and has no documented varicella vaccination. STIKO recommends individual catch-up vaccination to all children up to their 18th birthday and to all VZV-seronegative women of childbearing age.

We compared the detection of anti-VZV IgG by a commercially available ELISA with that by an in-house FAMA, which can be regarded as the gold standard [24]. We observed that 45.8% of samples with equivocal ELISA results were assessed as positive by FAMA. Additionally, 2.6% and 9.0%, respectively (depending on the age of the tested children), of ELISA-negative serum samples were determined to be positive by FAMA. As the gold standard assay, FAMA has a higher sensitivity and a lower detection limit than an ELISA [25]. The EUROIMMUN Anti-VZV (IgG) ELISA was reported to have a significantly lower sensitivity than FAMA, especially for the detection of anti-VZV IgG after varicella vaccination has been performed [14]. Interestingly, when serum samples that produced a negative or equivocal ELISA result were tested by FAMA, we more often observed a positive FAMA result in samples from varicella-vaccinated children than in samples from varicella-unvaccinated ones (Table 3). Although there was only a small number of varicella-vaccinated children in the study sample, our findings might be important for the monitoring of VZV seroprevalence in a varicella-vaccinated population. Because it is possible that anti-VZV IgG levels in varicella-vaccinated populations are overall lower, which would lead to a higher frequency of equivocal or even negative ELISA results, retesting these sera by FAMA might become much more relevant. Unfortunately, FAMA is a labor-intensive and time-consuming method that is not amenable to automation and requires experience for an appropriate interpretation of its results. Currently, FAMA tests are only performed in laboratories that are highly specialized in VZV diagnostics and research, and they are not commercially available. Therefore, it will be difficult to conduct FAMA for the detection of anti-VZV IgG on a routine basis.

Our study has several limitations. For the weighted seroprevalence rate calculation for varicella-unvaccinated children 1–17 years of age, we excluded 1156 study subjects with at least one reported varicella vaccination or with no vaccination card presented. Furthermore, most of the information on migration status, family members, and SES were self-reported or reported by the parents. However, this limitation would only influence the results of the risk factor analysis, not the VZV seroprevalence data.

## Conclusion

In our study, we determined the age-specific VZV seroprevalence among children and adolescents in the pre-varicella vaccine era in Germany. We confirmed that, in the absence of universal varicella vaccination, VZV seropositivity increased with age, especially in the first 6 years of life, which suggests that the major force of varicella transmission occurred in young children. We identified children without siblings in the same household and children with a migration background as being more susceptible to contracting varicella, even at higher ages (7–17 years old). Therefore, varicella-unvaccinated children or adolescents should be targeted for catch-up activities if they lack a history of chickenpox.

We also compared the test qualities of a commercially available ELISA with those of a gold standard test (FAMA). A high number of serum samples with a negative or equivocal result in the ELISA produced a positive FAMA result, especially those from varicella-vaccinated children. Our findings suggest that the commercially available ELISA can be used for the monitoring the VZV seroepidemiology in populations with or without an established universal varicella vaccination, but that FAMA testing is superior in situations with equivocal ELISA results or in ELISA-negative samples from individuals who were vaccinated against varicella in the past. This study provides baseline data on VZV seroepidemiology in children and adolescents for monitoring the impact of the recently implemented varicella vaccination strategy in Germany. Moreover, these data act as input for mathematical modelling of the varicella and herpes zoster-attributable disease burden and of future epidemiological effects of the varicella-vaccination [26].

## Abbreviations

CI: Confidence interval
ELISA: Enzyme-linked immunosorbent assay
FAMA: Fluorescent antibody to membrane antigen test
HSV: Herpes simplex virus
KiGGS: German Health Interview and Examination Survey for Children and Adolescents
OR: Odds ratio
SES: Social economic status
STIKO: German Standing Committee on Vaccination
VZV: Varicella-zoster virus
